# Seroprevalence of Hepatitis E Virus Infection in Middle Eastern Countries: A Systematic Review and Meta-Analysis

**DOI:** 10.3390/medicina58070905

**Published:** 2022-07-06

**Authors:** Fadi S. Qashqari

**Affiliations:** Department of Microbiology, College of Medicine, Umm Al-Qura University, Makkah 24381, Saudi Arabia; fsqashqari@uqu.edu.sa; Tel.: +966-553552660

**Keywords:** hepatitis E virus, prevalence, Middle Eastern countries, systematic review, meta-analysis

## Abstract

Hepatitis E virus (HEV) is a hepatotropic virus that is a major public health concern worldwide. Autochthonous HEV is spread through oral feces in unsanitary environments, as well as vertical and, occasionally, blood transfusion. HEV is more common in developing countries, but it has recently become more widespread in developed countries as well. The Middle East (ME) has long been an endemic location for HEV infection. Therefore, the aim of this systematic review and meta-analysis was to assess the seroprevalence of anti-HEV antibodies in ME countries. The author systematically searched five databases, namely ScienceDirect, EMBASE, Scopus, PubMed, and Google Scholar, to identify English-language articles published on or before 25 April 2022. Comprehensive meta-analysis software was used for all statistical analyses (CMA, version 3, BioStat, Englewood, CO, USA). After quality control and exclusion of irrelevant studies, 80 studies were included in the qualitative synthesis and meta-analysis. A forest plot showed that the overall pooled seroprevalence of HEV infection in ME countries in the fixed-effect and random-effect models were 21.3% (95% CI: 0.209–0.216) and 11.8% (95% CI: 0.099–0.144), respectively. Furthermore, the findings showed a high level of heterogeneity (I2 = 98.733%) among the included studies. In both fixed-effect and random-effect models, the seroprevalence of HEV infection by country was high in Egypt as compared to other regions, at 35.0% (95% CI: 0.342–0.359), and 34.7% (95% CI: 0.153–0.611), respectively. The seroprevalence of HEV infection by country was high among pregnant women, at 47.9% (95% CI: 0.459–0.499) in the fixed-effect model, and in renal transplant recipients, at 30.8% (95% CI: 0.222–0.410) in the random-effect model. The seroprevalence of HEV infection varies by country and study population in the Middle East. More research is needed to determine the disease’s incidence, morbidity, and mortality in the region, where it is prevalent.

## 1. Introduction

The World Health Organization (WHO) launched a global strategy to stop viral hepatitis transmission in 2016, recommending that persons with viral hepatitis have access to safe, accessible, and effective prevention, care, and treatment services [1]. By 2030, the goals are to reduce the number of new instances of hepatitis by 90 percent, treat 80 percent of eligible patients infected with viral hepatitis, and reduce the number of hepatitis-related fatalities by 65 percent [1]. Globally, nearly 1.34 million deaths were attributed to viral hepatitis in 2015, with 95 percent of those deaths attributed to chronic hepatitis B and C infections and the remainder to hepatitis A and E infections [1,2].

Global estimates suggest that more than 20 million new instances of hepatitis E virus (HEV) infections occur each year, with 3.3 million of those becoming symptomatic [3]. In 2015, the WHO reported 44,000 fatal HEV infections, accounting for about 3.3 percent of all viral hepatitis-related deaths [3].

HEV is a water- and food-borne illness that can cause severe epidemics in areas where sanitation is lacking [1,3]. However, there has been evidence of zoonotic and transfusion-related transmission [4,5]. Because there is no specific treatment for HEV infection, it is managed mostly through supportive care [1,3]. Prevention, on the other hand, focuses on limiting exposure through improved sanitation, clean food and drinking water, and vaccination [1]. In comparison to hepatitis B and C, HEV infection is less likely to cause chronic liver damage, and the development of fulminant hepatitis, albeit rare, is mostly influenced by host-specific rather than virus-specific variables [6].

Clinical signs and symptoms such as myalgia, arthralgia, anorexia, hepatomegaly, fever, weakness, vomiting, and jaundice emerge two to nine weeks after HEV exposure. In rare and severe cases, HEV can cause abrupt liver failure. Chronic instances are uncommon; however, they can occur in immunocompromised persons [7,8]. There is a variety of laboratory tests for HEV infection diagnosis, which can be divided into direct (detection of HEV or viral protein via polymerase chain reaction or enzyme immunoassay) and indirect (detection of anti-HEV antibodies) approaches [9,10]. Recent HEV infection is linked to the existence of IgM anti-HEV antibodies. Furthermore, the presence of anti-HEV IgG antibodies is indicative of recent or distant HEV exposure. Both antibodies are critical for HEV infection diagnosis and can be linked to long-term infection [9,10].

The majority of people in Middle Eastern (ME) countries live in middle-income countries, where viral hepatitis is a major health concern [11]. Furthermore, HEV infection is highly endemic in most of the countries in this region [12,13]. Given these countries’ changing socioeconomic conditions, identifying the epidemiological pattern of HEV infection will assist healthcare policymakers in making better decisions regarding future strategies for controlling this virus, as well as selecting and implementing cost-effective preventative methods [14,15].

Furthermore, to the very best of our knowledge, there remains a dearth of knowledge with respect to the prevalence of HEV-infected people with anti-HEV antibodies (IgG) in ME countries. Therefore, this systematic review and meta-analysis is the first attempt to provide a summarized and up-to-date estimation of the seroprevalence of HEV infection in ME countries. 

## 2. Materials and Methods

### 2.1. Data Sources and Literature Search Strategy

The author systematically searched five databases, namely ScienceDirect, EMBASE, Scopus, PubMed, and Google Scholar, to identify English-language articles published on or before 25 April 2022 that originally reported data on the prevalence of HEV infection in ME countries. The following keywords were used: “Hepatitis E virus”, “HEV”, and “Prevalence”, combined with the names of ME countries, namely Akrotiri and Dhekelia, Bahrain, Cyprus, Egypt, Iran, Iraq, Israel, Jordan, Kuwait, Lebanon, Oman, Palestine, Qatar, Saudi Arabia, Syria, Turkey, United Arab Emirates, and Yemen.

The current systematic review and meta-analysis was conducted according to the PRISMA recommendations (Supplementary Material S1) and was registered with the International Prospective Register of Systematic Reviews (PROSPERO, registration No. CRD42022330216).

### 2.2. Eligibility Criteria

The author included all observational studies conducted in ME countries that had, at least, an English abstract and reported on the prevalence of HEV-infected people with anti-HEV antibodies (IgG) among the general population, blood donors, hemodialysis patients, children, acute viral hepatitis patients, pregnant women, male blood donors, drug addicts, HIV positive individuals, thalassemia patients, soldiers, hemophiliac patients, renal transplant recipients, non-A-C hepatitis patients, and solid organ recipients. The systematic review and meta-analysis were designed to include people of all ages. Case reports, case series, letters, commentaries, editorials, non-human studies, symposia, correspondences, and citations without full text were all excluded from the study.

### 2.3. Study Screening and Data Extraction

The article screening process and removal of duplicates were managed using EndNote V.X8 software. Furthermore, two researchers (F.Q. and S.K.) meticulously and manually treated the data to reduce the chance of duplication.

The following details were extracted from the included articles using a standardized data collection form: first-author name, publication year, study sample, study country, sampling year, study population, type of study, participants’ age (range), study city, percentage of male participants, percentage of female participants, and prevalence of HEV-infected people with anti-HEV antibodies (IgG).

### 2.4. Quality Assessment

The quality of the included articles was assessed using the National Institute of Health quality assessment technique [16,17]. This assessment tool was used because it allows for a thorough evaluation of the quality of the research included. Furthermore, the general quality of the studies was graded as good, fair, or poor, and these ratings were incorporated into the meta-analytic results. The two researchers (F.Q. and R.A.) compared their evaluations for each study, and any disagreements were handled through a joint discussion.

### 2.5. Data Synthesis and Statistical Analysis

Comprehensive meta-analysis software was used for all statistical analyses (CMA, version 3, BioStat, USA). To reset the effect size value obtained from the meta-analysis, the fail-safe N approach was used to determine the number of studies that should be added to the meta-analysis. The average effect size of the meta-analysis studies was computed. The seroprevalence of HEV infection in ME countries was pooled and investigated using a random-effect model, with the results displayed in forest plots. Using the extracted data, the rate of events, their 95 percent confidence intervals, and their *p*-values were determined. The I^2^ statistic was used to assess the degree of heterogeneity among the included studies, with I^2^ values of 0–40%, 25–50%, 50–75%, and >75% indicating trivial, low, moderate, and high heterogeneity, respectively [18]. A non-significant degree of statistical heterogeneity was assumed when *p* < 0.1 or I^2^ < 50 percent [19]. Because of the considerable heterogeneity, a random-effects model was adopted. A funnel plot was used to discover potential signs of publication bias between included papers, as detected by Begg’s and Mazumdar’s rank correlation tests.

## 3. Results

### 3.1. Search Outcomes

**Figure 1:**
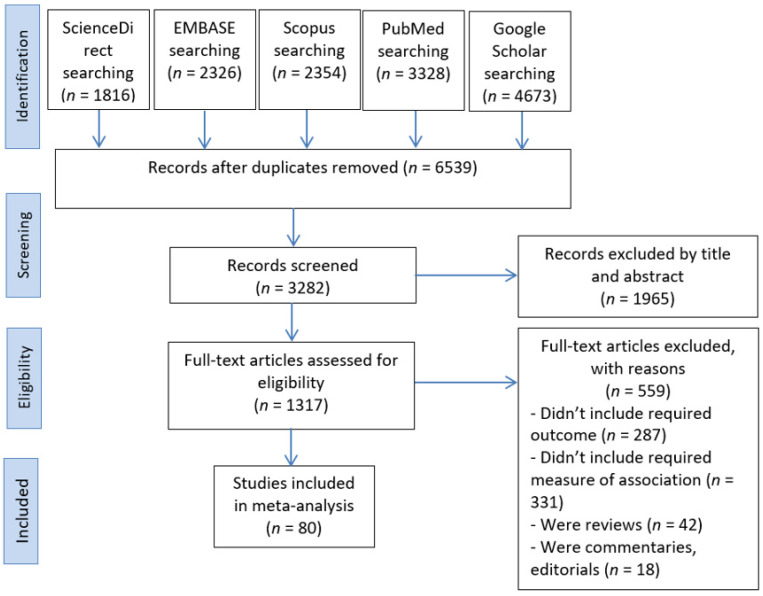
PRISMA flow chart of study identification and study selection process.

The search yielded a total of 14,497 articles from five databases: ScienceDirect (*n* = 1816), EMBASE (*n* = 2326), Scopus (*n* = 2354), PubMed (*n* = 3328), and Google Scholar (*n* = 4673). After duplicates were excluded, 6539 articles remained. A further 3257 articles were excluded due to the studies being conducted in non-ME countries, in addition to 1965 studies deemed irrelevant after screening the titles and abstracts. Then, we reviewed the full text of the remaining 1317 articles and excluded 1237 studies for not fulfilling our inclusion criteria. Ultimately, 80 studies were included in the qualitative synthesis and meta-analysis. The PRISMA flow chart for the process of article screening and selection is presented in Figure 1.

### 3.2. Characteristics of the Included Studies

Of the 80 included studies, 41 were conducted in Iran, 14 in Turkey, 8 in Egypt, 4 in Israel, 3 in Saudi Arabia, 3 in Iraq, 2 in Qatar, 1 in Kuwait, 1 in Syria, 1 in Yemen, 1 in the United Arab Emirates, 1 in Lebanon, 1 in Palestine, and 1 in Jordan. The prevalence of HEV IgG antibodies in the included studies ranged from 0.8% to 84.3% (range = 14.9). The targeted populations in the included studies were the general population (15 studies), blood donors (12 studies), hemodialysis patients (12 studies), children (11 studies), acute viral hepatitis patients (8 studies), pregnant women (7 studies), male blood donors (3 studies), drug addicts (3 studies), HIV-positive individuals (3 studies), thalassemia patients (2 studies), soldiers (1 study), hemophilia patients (1 study), renal transplant recipients (1 study), non-A-C hepatitis patients (1 study), and solid organ recipients (1 study). The sample size of the included articles ranged from 43 to 11,604 (average = 844) (Table 1).

### 3.3. Overall Pooled Seroprevalence of Hepatitis E Virus Infection in Middle Eastern Countries

**Figure 2:**
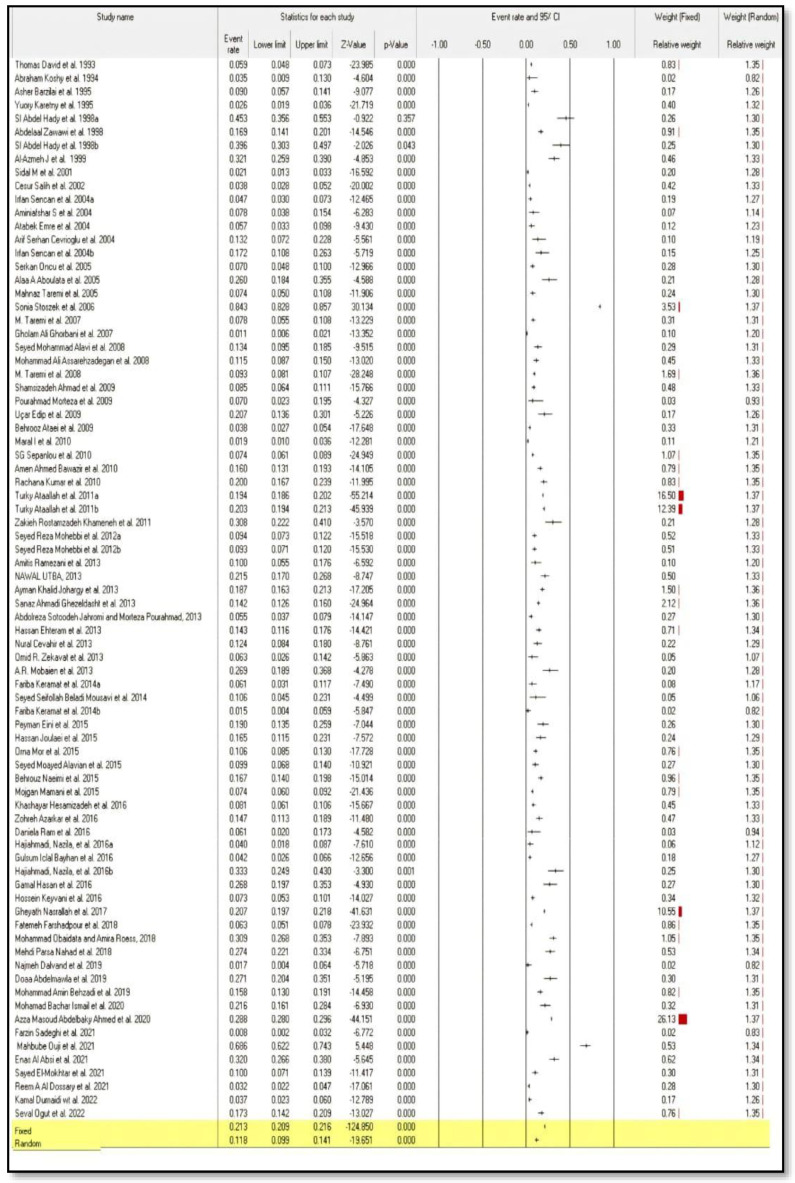
Forest plot meta-analysis of seroprevalence of hepatitis E virus infection in Middle Eastern countries [20,21,22,23,24,25,26,27,28,29,30,31,32,33,34,35,36,37,38,39,40,41,42,43,44,45,46,47,48,49,50,51,52,53,54,55,56,57,58,59,60,61,62,63,64,65,66,67,68,69,70,71,72,73,74,75,76,77,78,79,80,81,82,83,84,85,86,87,88,89,90,91,92,93].

All eighty included studies were pooled for meta-analysis; the forest plot showed that the overall pooled seroprevalence of HEV infection in ME countries in the fixed-effect and random-effect models was 21.3% (95% CI: 0.209–0.216), and 11.8% (95% CI: 0.099–0.144), respectively. Furthermore, the findings showed a high level of heterogeneity (I^2^ = 98.733%) among the included studies. Furthermore, the overall pooled seroprevalence of HEV infection in ME countries was statistically significant (pooled *p*-value < 0.001) in both fixed-effect and random-effect models (Figure 2). Table 2 shows the mean effect size and confidence intervals based on the random effect analysis of the studies in the meta-analysis.

### 3.4. Subgroup Analysis

**Figure 3:**
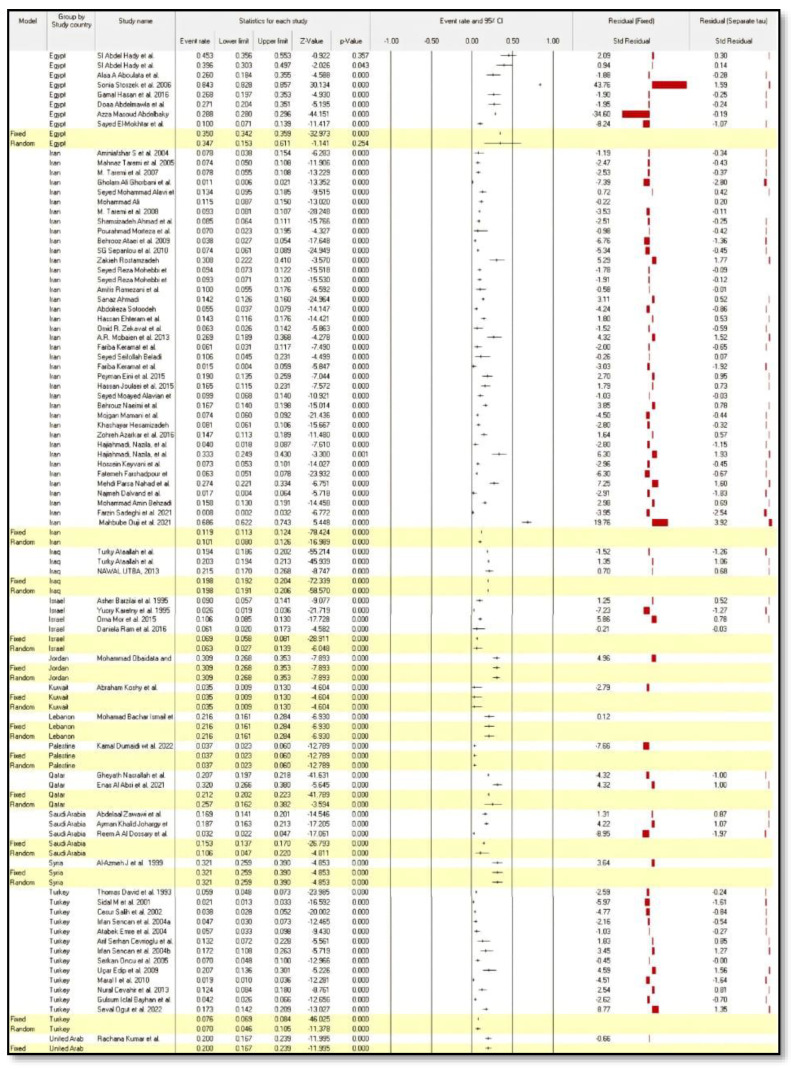
Forest plot meta-analysis of seroprevalence of hepatitis E virus infection by country [20,21,22,23,24,25,26,27,28,29,30,31,32,33,34,35,36,37,38,39,40,41,42,43,44,45,46,47,48,49,50,51,52,53,54,55,56,57,58,59,60,61,62,63,64,65,66,67,68,69,70,71,72,73,74,75,76,77,78,79,80,81,82,83,84,85,86,87,88,89,90,91,92,93].

In both fixed-effect and random-effect models, the seroprevalence of HEV infection by country was highest in Egypt as compared to other countries, at 35.0% (95% CI: 0.342–0.359) and 34.7% (95% CI: 0.153–0.611), respectively (Figure 3).

**Figure 4:**
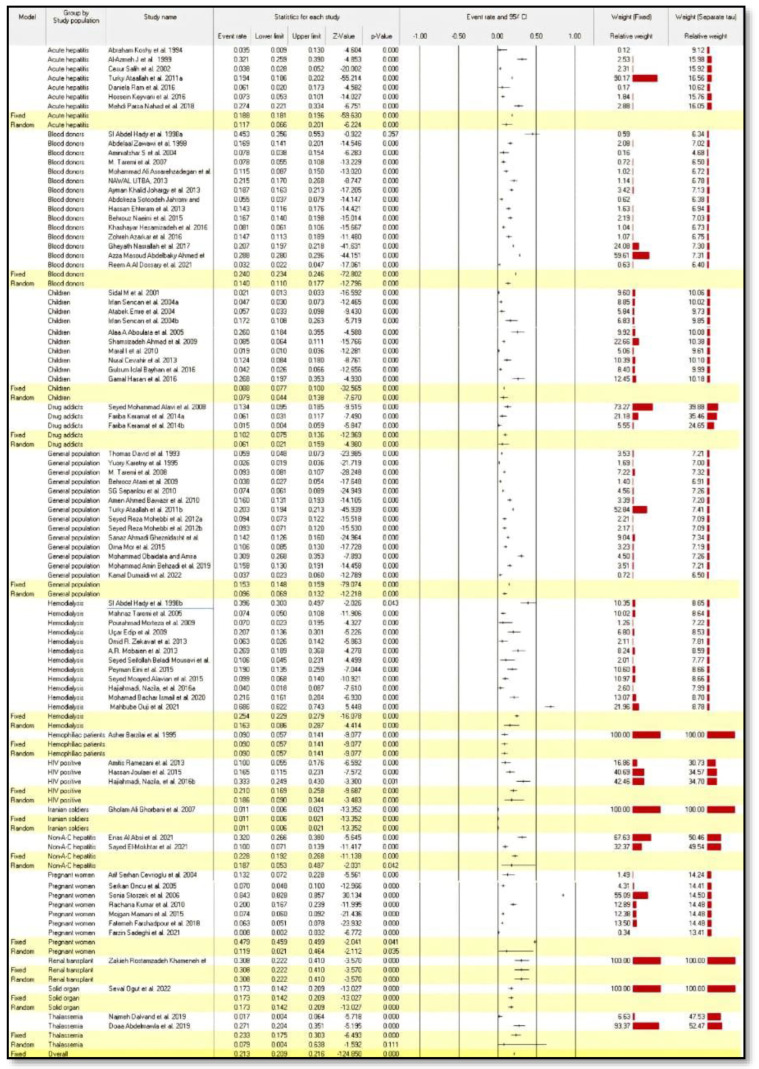
Forest plot meta-analysis of seroprevalence of hepatitis E virus infection by study population [20,21,22,23,24,25,26,27,28,29,30,31,32,33,34,35,36,37,38,39,40,41,42,43,44,45,46,47,48,49,50,51,52,53,54,55,56,57,58,59,60,61,62,63,64,65,66,67,68,69,70,71,72,73,74,75,76,77,78,79,80,81,82,83,84,85,86,87,88,89,90,91,92,93].

The seroprevalence of HEV infection by the study population was highest in pregnant women 47.9% (95% CI: 0.459–0.499) in the fixed-effect model and in renal transplant recipients, 30.8% (95% CI: 0.222–0.410) in the random-effect model, as compared to other populations (Figure 4).

### 3.5. Publication Bias

**Figure 5:**
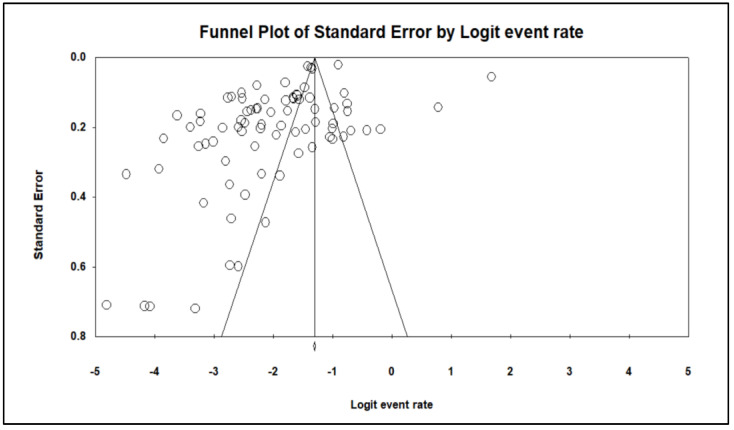
Publication bias of the seroprevalence of hepatitis E virus infection in Middle Eastern countries.

The results of Begg’s and Mazumdar’s rank correlation tests revealed a dispersed distribution, implying publication bias. The *p*-values for Kendall’s tau without continuity and Kendall’s tau with continuity were both 0.001 (Table 3). Figure 5 depicts a funnel plot of the seroprevalence of HEV infection in ME countries with publication bias.

## 4. Discussion

Worldwide, seroprevalence-based studies have received increased attention in recent years. However, due to incorrect diagnosis, underestimation, and a lack of awareness among clinicians about HEV, the published literature contains considerable gaps [94,95]. As a result, the goal of this study was to determine the seroprevalence of HEV infection in ME countries. Researchers and policymakers may benefit from the information in this systematic review and meta-analysis in order to better understand disease spread and develop effective control and prevention methods, particularly in ME countries.

Our findings showed that the seroprevalence of HEV infection in ME countries ranged from 0.8% among Iranian pregnant women [88] to 84.3% among Egyptian pregnant women [37]. Different test methodologies and geographic locations, research sample size, surveillance year, and other factors could explain these observed differences in HEV seroprevalence. However, in our study, in the fixed-effect and random-effect models, the overall pooled seroprevalence of HEV infection in ME countries was 21.3% and 11.8%, respectively. According to a recent systematic review and meta-analysis, the overall pooled prevalence of HEV infection in pregnant women around the world was 16.51% [95]. A systematic review of HEV seroprevalence in thirteen African nations found that it ranged from zero to eighty-four percent, with pregnant women and rural areas having higher immunoglobulin levels than other areas [96]. The estimated pooled seroprevalence of HEV in Chinese blood donors was 30%. In European countries, the estimated seroprevalence of HEV ranged from 0.6% to 52.5% [97]. Another comprehensive evaluation of the Brazilian population found a 6.0% overall seroprevalence of HEV infection [98]. Furthermore, the pooled prevalence in our study is higher than in certain primary studies conducted among pregnant women in different countries, such as Serbian blood donors (15.0%) [99], as well as in Mexico (5.7%) [100], Pakistan (8.86%) [101], and Sudan (10.3%) [102].

Our analysis showed that Egypt has the highest seroprevalence of HEV infections compared to other countries. In Egypt, HEV infection is a neglected disease. In Egyptian hospitals, HEV testing is not frequently used for the diagnosis of suspected hepatitis patients [90]. Anti-HEV IgG seroprevalence in Egyptians is among the highest in the world, at up to 84 percent [37,103]. Furthermore, an HEV outbreak was previously observed in Assiut governorate rural villages [104].

Despite the fact that the incidence of HEV has declined significantly in recent years as a result of improved hygiene conditions [105], our analysis revealed that the seroprevalence of HEV infection was higher in pregnant women when compared to other populations. There is a considerable chance of vertical transmission of these viruses from the mother to the fetus, which can result in maternal and fetal problems, such as abortion, neonatal mortality, and early labor [106]. To avoid any negative consequences, it is critical to diagnose HEV infections in pregnant women.

An increasing number scientists and researchers are becoming aware of the repercussions of HEV infection, which include severe liver impairment and a high rate of morbidity and mortality, particularly in pregnant women. As a result, the pathophysiology and immunology of HEV interaction during pregnancy have received increased attention. However, especially in HEV endemic areas, it is critical to investigate genetic and environmental causes. To control and stop the disease in the near future, immunological research and prevention, as well as treatment measures, must be enhanced. In addition, in countries where the disease is endemic, cost-effective immunization efforts are required.

Despite the fact that the current meta-analysis has a large sample size and includes all ME countries and populations previously researched, it is subject to numerous limitations. The majority of the studies reviewed used distinct anti-HIV IgG ELISA kits with varying specificity and sensitivity, which could impair the reliability and accuracy of the tests. Only the anti-HEV IgG antibody level, which appears mainly after infection, was used to determine seroprevalence. Furthermore, the studies included in this systematic review and meta-analysis were observational, with a wide range of baseline characteristics, sample sizes, and sampling years.

## 5. Conclusions

The seroprevalence of HEV infection varies by country and study population in the ME and is highest in Egypt as compared to other countries and is highest in pregnant women and in renal transplant recipients as compared to other populations. More research is needed to determine the disease’s incidence, morbidity, and mortality in the region, where it is prevalent. In addition, essential steps should be taken to control and prevent HEV infection in general and in pregnant women in particular. Visiting endemic areas requires extra attention, especially when it comes to drinking water and food safety.

## Figures and Tables

**Table 1:** Characteristics of the included studies in the systematic review and meta-analysis.

First-Author Name	Publication Year	Study Sample	Study Country	Sampling Year	Study Population	Type of Study	Participant Age (Range)	Study City	Male (%)	Female (%)	Prevalence (%)	Ref.
Thomas David	1993	1350	Turkey	1990–1992	General population	Cross-sectional	18–65 years	Istanbul, Ayvalik, Aydin, Trabzon region, and Adana	50.2	49.8	5·9	[20]
Abraham Koshy	1994	57	Kuwait	1992	Acute viral hepatitis patients	Cross-sectional	19–46 years	Kuwait	88	12	4	[21]
Asher Barzilai	1995	188	Israel	NM	Hemophiliac patients	Cross-sectional	2–75 years	Tel Aviv	98.9	1.1	9	[22]
Yuory Karetny	1995	1416	Israel	1988–1993	General population	Cross-sectional	1–66 years	West Bank and central region of Israel	NM	NM	2.6	[23]
Abdelaal Zawawi	1998	593	Saudi Arabia	1995	Male blood donors	Cross-sectional	15–60 years	Jeddah	100	0	16.9	[24]
SI Abdel Hady	1998	95	Egypt	NM	Blood donors	Cross-sectional	NM	NM	NM	NM	45.2	[25]
SI Abdel Hady	1998	96	Egypt	NM	Hemodialysis patients	Cross-sectional	NM	NM	NM	NM	39.6	[25]
Al-Azmeh J	1999	193	Syria	1995–1998	Acute hepatitis patients	Hospital-based	12–70 years	Damascus	52.4	47.6	31.9	[26]
Sıdal M	2001	909	Turkey	1997–1998	Children	Cross-sectional	Six months–15 years	Istanbul	NM	NM	2.1	[27]
Colak D	2002	338	Turkey	1996–1997	Pediatric age groups	Cross-sectional	1–11 years	Antalya	NM	NM	0.89	[28]
Cesur Salih	2002	1046	Turkey	2000–2001	Adults	Cross-sectional	15–75 years	Ankara	NM	NM	3.8	[29]
Arif Serhan Cevrioglu	2004	76	Turkey	2000–2002	Pregnant women	Cross-sectional	19–42 years	Afyon	0	100	12.6	[30]
Irfan Sencan	2004	383	Turkey	1999	Children	Cross-sectional	2–15 years	Du¨zce	51.7	48.3	4.7	[31]
Atabek Emre	2004	210	Turkey	2001–2002	Children	Cross-sectional	1–18 years	Konya	49	51	5.5	[32]
Aminiafshar S	2004	90	Iran	2003–2004	Blood donors	Cross-sectional	40–49 years	Tehran	80.2	19.8	7.8	[33]
Irfan Sencan	2004	93	Turkey	1999	Children	Cross-sectional	2–15 years	Golyaka	37.6	62.4	17.2	[31]
Serkan Oncu	2005	386	Turkey	NM	Pregnant women	Cross-sectional	18–32 years	Aydin	0	100	7	[34]
Alaa A Aboulata	2005	100	Egypt	2004–2005	Children presenting with minor hepatic disorders	Cross-sectional	1–10 years	Cairo	NM	NM	26	[35]
Mahnaz Taremi	2005	324	Iran	2004	Hemodialysis patients	Cross-sectional	18–80 years	Tabriz	59	41	7.4	[36]
Sonia Stoszek	2006	2428	Egypt	1997–2003	Pregnant women	Cross-sectional	18–40 years	Nile Delta	NM	NM	84.3	[37]
M. Taremi	2007	399	Iran	2004	Male blood donors	Cross-sectional	20–60 years	Tabriz	100	0	7.8	[38]
Gholam Ali Ghorbani	2007	800	Iran	2006	Soldiers	Cross-sectional	17–23 years	Tehran	100	0	1.1	[39]
Seyed Mohammad Alavi	2008	224	Iran	2005–2006	Drug addicts	Cross-sectional	18–54 years	Ahvaz	100	0	13.5	[40]
Mohammad Ali Assarehzadegan	2008	400	Iran	2005	Blood donors	Cross-sectional	18–60 years	Khuzestan	65	35	11.5	[41]
M. Taremi	2008	1824	Iran	2003	General population	Cross-sectional	6–80 years	Nahavand	NM	NM	9.3	[42]
Uçar Edip	2009	92	Turkey	NM	Hemodialysis patients	Cross-sectional	22–71 years	Hatay	58.7	41.3	20.6	[43]
Shamsizadeh Ahmad	2009	566	Iran	2006–2007	Children	Cross-sectional	6–15 years	Southwestern Iran	45.4	54.6	8.5	[44]
Behrooz Ataei	2009	816	Iran	2005	General population	Cross-sectional	6–60 years	Isfahan	47.5	52.5	3.8	[45]
Pourahmad Morteza	2009	43	Iran	2007	Hemodialysis patients	Cross-sectional	NM	Jahrom	67.4	32.6	7	[46]
Maral I	2010	515	Turkey	2003–2005	Primary school children	Cross-sectional	6–13 years	Ankara	52.7	47.3	1.9	[47]
Amen Ahmed Bawazir	2010	538	Yemen	2005	General population	Cross-sectional	one month–79 years	Aden	52	48	16	[48]
Rachana Kumar	2010	469	United Arab Emirates	NM	Pregnant women	Cohort	NM	Al Ain	0	100	20	[49]
SG Sepanlou	2010	1423	Iran	2009	General population	Cross-sectional	NM	Tehran and Golestan	NM	NM	7.4	[50]
Turky Ataallah	2011	9610	Iraq	2005–2006	Acute viral hepatitis	Cross-sectional	1–60 years	Baghdad	49.5	50.5	19.4	[51]
Turky Ataallah	2011	6972	Iraq	2005–2006	General population	Cross-sectional	1–60 years	Baghdad	48.8	51.2	20.3	[51]
Zakieh Rostamzadeh Khameneh	2011	91	Iran	NM	Renal transplant recipients	Cross-sectional	6–65 years	Urmia	67	33	30.8	[52]
Seyed Reza Mohebbi	2012	551	Iran	2006–2007	General population	Cross-sectional	1–83 years	Tehran	36.3	63.7	9.4	[53]
Seyed Reza Mohebbi	2012	551	Iran	2006–2007	General population	Cross-sectional	1–83 years	Tehran	50	50	9.3	[53]
Abdolreza Sotoodeh Jahromi	2013	477	Iran	2009	Blood donors	Cross-sectional	17–59 years	Jahrom	447	30	5.4	[54]
Sanaz Ahmadi Ghezeldasht	2013	1582	Iran	2012	General population	Cross-sectional	1–90 years	Mashhad	45.4	54.6	14.2	[55]
Nural Cevahir	2013	185	Turkey	NM	Primary school children	Cross-sectional	7–14 years	Denizli	50.3	49.7	12.4	[56]
Hassan Ehteram	2013	530	Iran	2012	Blood donors	Cross-sectional	31–50 years	Central province	91.9	8.1	14.3	[57]
Omid Zekavat	2013	80	Iran	2010	Patients with chronic maintenance hemodialysis	Cross-sectional	26–80 years	Southwestern Iran	63.7	63.3	6.3	[58]
A.R. Mobaien	2013	93	Iran	2011	Hemodialysis patients	Cross-sectional	16–88 years	Tehran	52.7	47.3	26.9	[59]
Ayman Khalid Johargy	2013	900	Saudi Arabia	2009	Male blood donors	Cross-sectional	18–66 years	Makkah	100	0	18.7	[60]
Nawal Utba	2013	270	Iraq	NM	Blood donors and cleaning workers	Cross-sectional	18–60 years	Baghdad	67	33	21.5	[61]
Amitis Ramezani	2013	100	Iran	2012	HIV-positive individuals	Cross-sectional	34–43 years	Tehran	71	29	10	[62]
Fariba Keramat	2014	131	Iran	2011–2012	Injection drug users	Cross-sectional	22–70 years	Hamadan	99.2	0.8	6.1	[63]
Fariba Keramat	2014	131	Iran	2011–2012	Non-injection drug users	Cross-sectional	20–45 years	Hamadan	99.2	0.8	1.5	[63]
Seyed Seifollah Beladi Mousavi	2014	47	Iran	NM	Hemodialysis patients	Cross-sectional	20–80 years	Ahvaz	57.4	42.6	10.6	[64]
Peyman Eini	2015	153	Iran	2010	Hemodialysis patients	Cross-sectional	10–70 years	Hamadan	54.2	45.8	19.2	[65]
Orna Mor	2015	729	Israel	2009–2010	General population	Cross-sectional	10–75 years	Tel-Aviv	54	46	10.6	[66]
Mojgan Mamani	2015	1050	Iran	2010–2012	Pregnant women	Prospective cross-sectional	14–49 years	Hamadan	0	100	7.4	[67]
Seyed Moayed Alavian	2015	274	Iran	2012	Hemodialysis patients	Cross-sectional	21–80 years	Isfahan	52.9	47.1	9.9	[68]
Hassan Joulaei	2015	158	Iran	2012–2013	HIV-positive individuals	Cross-sectional	1–60 years	Shiraz	76.9	23.1	16.4	[69]
Behrouz Naeimi	2015	628	Iran	2013	Blood donors	Cross-sectional	19–65 years	Bushehr	95.2	4.8	16.7	[70]
Daniela Ram	2016	49	Israel	2013–2015	Acute hepatitis patients	Cross-sectional	NM	Haifa, Tel Aviv, Beer Sheva	NM	NM	6.1	[71]
Hossein Keyvani	2016	200	Iran	NM	Blood donors	Cross-sectional	20–61 years	Tehran	58.2	41.8	4.5	[72]
Hossein Keyvani	2016	100	Iran	NM	Patients with hepatitis C	Cross-sectional	20–61 years	Tehran	58.2	41.8	7	[72]
Hossein Keyvani	2016	150	Iran	NM	Patients with hepatitis B	Cross-sectional	20–61 years	Tehran	58.2	41.8	11.3	[72]
Hajiahmadi Nazila	2016	149	Iran	NM	Hemodialysis patients	Cross-sectional	15–90 years	Golestan	49	51	4	[73]
Hajiahmadi Nazila	2016	102	Iran	NM	HIV-infected patients	Cross-sectional	17–54 years	Golestan	68.6	31.4	33.3	[73]
Khashayar Hesamizadeh	2016	559	Iran	2014	Blood donors	Cross-sectional	18–37 years	Tehran	95.9	4.1	8.1	[74]
Zohreh Azarkar	2016	340	Iran	2013–2014	Blood donors	Cross-sectional	20–40 years	Birjand	93.8	2.2	14.7	[75]
Gamal Hasan	2016	123	Egypt	2007–2008	Children	Multicenter prospective	2–18 years	Assiut	59.3	40.7	26.8	[76]
Gülsüm İclal Bayhan	2016	408	Turkey	2014	Children	Cross-sectional	2 months-18 years	Van	43.9	56.1	4.2	[77]
Gheyath Nasrallah	2017	5854	Qatar	2013–2016	Blood donors	Cross-sectional	15–80 years	Al Doha	97.4	2.6	20.7	[78]
Mohammad Obaidata	2018	450	Jordan	2015–2016	Patients who visit healthcare clinics for routine care	Cross-sectional	20–80 years	Eight governorates	45.1	54.9	30.9	[79]
Fatemeh Farshadpour	2018	1331	Iran	2016–2017	Pregnant women	Cross-sectional	14–45 years	Bushehr	0	100	6.3	[80]
Mehdi Parsa Nahad	2018	241	Iran	2013–2016	Acute viral hepatitis patients	Cross-sectional	10–80 years	Ahvaz	51.9	48.1	27.4	[81]
Najmeh Dalvand	2019	120	Iran	2019	Thalassemia-positive patients	Cross-sectional	17–45 years	Tehran	35	65	1.67	[82]
Mohammad Amin Behzadi	2019	562	Iran	2016–2017	Healthy individuals	Cross-sectional	1–86 years	Hormozgan	29.2	70.8	15.8	[83]
Doaa Abdelmawla	2019	140	Egypt	2016	Children with transfusion-dependent thalassemia	Cross-sectional	2–6 years	Mansoura	47.1	52.9	27.15	[84]
Mohamad Bachar Ismail	2020	171	Lebanon	2016	Hemodialysis patients	Cross-sectional	23–82 years	Tripoli	43.8	56.2	21.63	[85]
Azza Masoud Abdelbaky Ahmed	2020	11,604	Egypt	2013–2014	Blood donors	Cross-sectional	18–60 years	Qena	88.2	11.8	28.8	[86]
Mahbube Ouji	2021	226	Iran	NM	Hemodialysis patients	Cross-sectional	23–87 years	Bushehr, Borazjan, and Genaveh	56.2	43.8	68.6	[87]
Farzin Sadeghi	2021	247	Iran	2020	Pregnant women	Cross-sectional	17–42 years	Northern Iran	0	100	0.8	[88]
Reem A Al Dossary	2021	806	Saudi Arabia	2020	Blood donors	Cross-sectional	18–85 years	Eastern province	94.9	5.1	3.2	[89]
Sayed El-Mokhtar	2021	300	Egypt	2016–2018	Non-A-C hepatitis patients	Cross-sectional	40–60 years	Assiut	53	47	10	[90]
Enas Al Absi	2021	259	Qatar	2017–2019	Non-A-C hepatitis patients	Cross-sectional	6–98 years	Al Doha	61.4	83.6	32.1	[91]
Kamal Dumaidi	2022	432	Palestine	2015–2017	General population	Cross-sectional	1–86 years	West Bank and Jerusalem	49.3	50.7	3.7	[92]
Seval Öğüt	2022	485	Turkey	NM	Solid organ recipients	Cross-sectional	1–80 years	Izmir	64.7	35.3	17.3	[93]

NM denotes “not mentioned”.

**Table 2:** Meta-analysis and effect analysis values of included studies, homogeneous distribution value, average effect size, and confidence intervals.

Model	Effect Size and 95% Confidence Interval				Test of Null (2-Tail)		Heterogeneity				Tau-Squared			
Model	Number of Studies	Point of Estimate	Lower Limit	Upper Limit	Z-Value	*p*-Value	Q-Value	df (Q)	*p*-Value	I Squared	Tau Squared	Standard Error	Variance	Tau
Fixed	80	0.213	0.216	0.293	−124.850	0.000	6154.911	79	0.000	98.733	0.763	0.372	0.138	0.874
Random	80	0.118	0.141	0.253	−19.651	0.000								

**Table 3:** Begg’s and Mazumdar’s rank correlation.

Kendall’s S Statistic (P-Q)	6154.911
Kendall’s tau without continuity correction	
Tau	0.7633
z-value for tau	−124.850
*p*-value (1-tailed)	0.001
*p*-value (2-tailed)	0.001
Kendall’s tau with continuity correction	
Tau	0.8737
z-value for tau	−19.65
*p*-value (1-tailed)	0.001
*p*-value (2-tailed)	0.001

## Data Availability

Not applicable.

## Supplementary Materials

The following supporting information can be downloaded at: https://www.mdpi.com/article/10.3390/medicina58070905/s1, Supplementary Material S1: The PRISMA recommendations checklist [107].
